# Parental health limitations, caregiving and loneliness among women with widowed parents: longitudinal evidence from France

**DOI:** 10.1007/s10433-018-0459-2

**Published:** 2018-02-12

**Authors:** Thijs van den Broek, Emily Grundy

**Affiliations:** 1 London School of Economics and Political Science, London, UK; 2 University of Essex, Colchester, UK

**Keywords:** Intergenerational support, Wellbeing, Mental health, Linked lives, Life course, Fixed effects

## Abstract

We investigate how daughters’ feelings of loneliness are impacted when widowed parents develop health limitations, and when daughters take on personal care tasks in response. Using longitudinal data from daughters of widowed parents drawn from the French Family and Intergenerational Relationships Study (ERFI, 1485 observations nested in 557 daughters), we assess (a) whether health limitations of widowed parents are associated with daughters’ feelings of loneliness regardless of whether or not daughters provide personal care and (b) whether there is an effect of care provision on loneliness that cannot be explained by parental health limitations. Fixed effect regression analyses show that widowed parents’ health limitations were associated with raised feelings of loneliness among their daughters. No significant additional effect of providing personal care to a widowed parent was found. Prior research on the impact of health limitations of older parents on the lives of their adult–children has focused mostly on issues related to informal caregiving. Our findings suggest that more attention to the psychosocial impact of parental health limitations—net of actual caregiving—on adult children’s lives is warranted.

**Electronic supplementary material:**

The online version of this article (10.1007/s10433-018-0459-2) contains supplementary material, which is available to authorized users.

## Introduction

Loneliness is the unpleasant experience of perceiving one’s personal network of social relations as being insufficiently supportive (Peplau and Perlman 1982). It has been identified as a risk factor for poor physical and mental health and mortality (Hawkley and Cacioppo 2010; Ong et al. 2016). Research on factors associated with loneliness has mainly focused on the role of personal characteristics and circumstances, such as gender, age, marital and employment status, health, and socio-economic resources (Ong et al. 2016; Pinquart and Sörensen 2001; Routasalo and Pitkala 2003). However, as noted by Elder (1985), within families, “each generation is bound to […] events in the other’s life course” (p. 40). Building on this notion, we use longitudinal French data to investigate firstly how daughters’ loneliness is impacted when a widowed parent develops health limitations, and secondly the impact on daughters’ loneliness of providing personal care for a widowed parent.

As discussed in further detail below, having a parent with health limitations and provision of care to ageing parents may both be expected to have psychosocial consequences for adult children. Given that parental health limitations and adult children’s care provision are closely intertwined (Walker et al. 1995), studies estimating the psychosocial impact of the former should take the latter into account as well and vice versa (cf. Amirkhanyan and Wolf 2003, 2006; Wolf, Raissian, and Grundy 2015). The goal of the current study is therefore twofold. We aim to assess (a) whether health limitations of widowed parents are associated with daughters’ feelings of loneliness regardless of whether or not daughters provide personal care and (b) whether there is an additional effect of care provision on loneliness.

In pursuit of answers to these research questions, we analyse longitudinal French data from the Family and Intergenerational Relationships Study. We focus specifically on adult daughters with a widowed parent. Our choice to focus on daughters is largely pragmatic. Daughters are known to be favoured over sons as a source of support by ageing parents (Suitor and Pillemer 2006), and older people in France are more than twice as likely to receive support from daughters as from sons (Attias-Donfut 2001). Consistent with these findings, preliminary analyses showed that in our data very few sons provided care to ageing parents, making it unfeasible to include them, given the twofold research aim described above.

The decision to focus only on children of widowed persons was theoretically driven. Litwak’s (1985) task specificity model holds that support for older persons in need tends to be provided by the available source whose characteristics best match what is needed for the support task at hand. Provision of personal care requires long-term commitment and continuous proximity, and spouses are the most likely people to meet these criteria (Messeri et al. 1993). Consequently, adult children will be considered first in line to take on care tasks only when a spouse or partner is not available, for instance due to widowhood (cf. Shanas 1979). Consistent with this reasoning, Ha et al. (2006) found that older persons’ dependency on their adult children grows with widowhood and Blomgren et al. (2012), among others, have shown that ageing parents living with a spouse are markedly less likely to receive support from their adult children than their counterparts not living with a spouse (cf. Jacobs et al. 2016; Van den Broek et al. 2017). Arguably, the experience of providing care for a widowed parent is also qualitatively different from providing care to a parent with a spouse or partner as in the former case the adult child may be the sole or major care provider, whereas in the latter case the caring responsibility may be a subsidiary one. Caregiving adult children are known to spend less time on support for their ageing parents when the latter are still living with a spouse (Stoller 1983). In considering daughters of widowed parents, we thus focus on a group that is not only relatively likely to be caregivers, but also a group for whom the psychosocial consequences of caring for a parent with functional limitations may be especially strong.

## Theoretical background and hypotheses

The premise of the current study is the notion from the life course perspective that human lives are linked, i.e. interdependent as a consequence of their embeddedness in social relationships with kin and friends across the lifespan (Elder 1994). When older persons develop health limitations, plausibly not only their own lives, but also those of persons close to them are affected. Parental health limitations may diminish the amount of support that adult children can derive from their personal networks of social relations and increase the need for such support, both directly and indirectly through adult children’s tendency to take on care tasks in response to parental health limitations.

We have not been able to identify any previous studies on the association between parents’ health limitations and adult children’s feelings of loneliness, and the body of research on loneliness among adult children who provide care to ageing parents is small and inconclusive. Beeson et al. (2000) and McRae et al. (2009) analysed cross-sectional samples of caregivers in the USA and found that levels of loneliness were relatively high. The latter compared levels of loneliness among caregivers from these two studies to levels of loneliness found in samples of non-caregiving persons in the same age range and concluded that levels of loneliness were higher among caregivers. In contrast, Hansen et al. (2013), drawing on Norwegian data, found little evidence that caring for a parent was associated with loneliness (cf. Hansen and Slagsvold 2015). Only women who provided care to co-resident parents had raised feelings of loneliness.

The small body of research on the links between caregiving to parents and loneliness does not include any studies exploring the association between parents’ health limitations—net of actual caregiving—and feelings of loneliness among their children. Hansen et al. (2013), who found an association between provision of personal care to a co-resident parent and loneliness among Norwegian women, noted that “it is unclear whether it is the caregiving itself or the fact that a close […] relative is frail that may harm women’s wellbeing” (p. 339).

### Parental health limitations and loneliness

Parents are an important source of support for their children throughout the life course. Even at older ages parents provide more support to their children than vice versa, but parents with health limitations are less likely to provide support to children (Albertini et al. 2007). Hence, when parents develop health limitations, this may involve a reduction in the support that adult children receive from parents, and so an overall reduction in available support. Any resulting mismatch in availability of and need for social support may result in loneliness.

Parental health limitations may also be a source of stress for adult children, regardless of whether or not they provide care (Amirkhanyan and Wolf 2003, 2006; Wolf et al. 2015). Stress can, in turn, result in increased feelings of loneliness (Aanes et al. 2010, 2011). As a consequence of stress associated with the experience of having a parent with health limitations, children may have an increased need and desire for a supportive social network. When their actual networks are insufficiently supportive to meet this need, feelings of loneliness may arise. This is illustrated by the following quote from a daughter of a dying mother, taken from a qualitative study of Canadian women by Read and Wuest (2007):

My brother phoned one night and I told him, “Mom is in the hospital and she has pneumonia.” I practically cried on the phone and I said, “Look, I’m having a real hard time.” He said, ‘Life is hard anyway.’ I felt so alone. There was just me. (p. 937)

This suggests that adult daughters may be expected to be at increased risk of evaluating their personal network of social relationships as insufficiently supportive when parents develop health limitations. We therefore expect that parental health limitations are associated with raised feelings of loneliness among their daughters (Hypothesis 1).

### Caring for parents and loneliness

Children may respond to parental health limitations by taking on support tasks (Blomgren et al. 2012; Silverstein et al. 2006; Van den Broek and Dykstra 2017). Although providing informal care can be rewarding (Cohen et al. 2002; Habermann et al. 2013), it can also be demanding and time consuming (Attias-Donfut 2001; Habermann et al. 2013). This is particularly the case when personal care, e.g. help with dressing or bathing, is provided, given the intensity of this type of support (Walker et al. 1995). Qualitative studies suggest that caregivers often have difficulties finding time for family members, friends and colleagues, and have problems establishing and maintaining relationships (Hawranik and Strain 2007; McDonnell and Ryan 2013; cf. Pearlin et al. 1990). Many caregivers express fear of social isolation (Kenny et al. 2012; Stoltz et al. 2004). Declines in engagement in social activities have been identified as risk factors for loneliness (Aartsen and Jylhä 2011; Dahlberg et al. 2016), which implies that when caregiving demands result in neglect of the social network, this may lead to loneliness.

In addition to the risk that caregiving may result in deterioration of the quality of adult children’s social networks, caregiving may also increase needs for support from one’s social network. Social support derived from one’s network may buffer stress and mitigate any detrimental effects of caregiving on wellbeing (Pearlin et al. 1990). Qualitative studies indicate that children taking on the role of caregiver for an impaired parent often find that the extent to which they can count on their social network—and in particular on their siblings—is below what they expected (Amaro and Miller 2016; Kenny et al. 2012; Merrill 1996; Neufeld and Harrison 2003). Donorfio and Kellett (2006) found that daughters who cared for frail mothers often felt isolated with no one to turn to for counsel or advice. Merrill (1996) described the disappointment and the deterioration of the relationship with her siblings of an American woman who tried for over a year to get her brother and sister, both of whom lived nearby, to occasionally take care of their frail mother:

I really thought that my brother and sister would do their part, no questions. My brother and I used to do things together, now we are farther apart than ever. (p. 408)

As this suggests, when parents with health limitations are cared for by their children, this may be source of conflict between siblings (Suitor et al. 2014), and adults who have conflicts with siblings are lonelier than their counterparts with harmonious sibling relationships (Ponzetti and James 1997). For all these reasons, we expect that the provision of personal care to a widowed parent will be associated with raised feelings of loneliness among daughters (Hypothesis 2).

## Data and methods

### Data

We use longitudinal data from the *Etude des relations familiales et intergénérationnelles* (Family and Intergenerational Relationships Study, ERFI) (Régnier-Loilier 2016). ERFI is the French component of the Generations and Gender Surveys (cf. Vikat et al. 2007). It is based on a two-stage sample design, whereby areas were selected in the first stage, followed by a selection of dwellings from the census. Baseline and follow-up data collection took place in 2005, 2008 and 2011, respectively. The response rate for the first wave was 65%, and attrition was 35% between waves 1 and 2 and 17% between waves 2 and 3. To account for selective initial non-response and subsequent attrition, ERFI provides longitudinal weights that make the data representative of the French population in 2005 with regard to gender and age group, number of people living in the dwelling, occupational category, nationality, level of urbanization, and region of residence (Régnier-Loilier 2016; Régnier-Loilier and Guisse 2016). In this article, we present results of analyses of weighted data. Results of the analyses of unweighted data—which were very similar to the results presented here—are available as an online supplement.

We limited our sample to 563 women for whom at least two observations were available at which their mother or father was widowed. Respondents were not directly asked for the marital status of their parents, but we were able to infer widowhood status of a parent from the daughter’s report that the other parent was dead, that the parents had never separated, and that the surviving parent was not living with a new partner or spouse. Information on whether or not the surviving parent was living with a new partner or spouse was not available in Wave 1. When a respondent’s surviving parent met all other widowhood criteria in Wave 1 and was not living with a new partner or spouse in the next available observation, we assumed that the parent was not living with a new partner or spouse in Wave 1 either and coded the parent as widowed. After dropping observations with missing values on any variable of interest, a final sample of 1485 observations for 557 women remained.

### Measures

#### Outcome variable

Loneliness was measured using the shortened De Jong Gierveld loneliness scale (De Jong Gierveld and Van Tilburg 2006), which contains three negatively formulated items (“I experience a general sense of emptiness”, “I miss having people around”, and “Often, I feel rejected”) and three positively formulated items (“There are plenty of people that I can lean on in case of trouble”, “There are many people that I can count on completely”, and “There are enough people that I feel close to”), all of which have response categories of “yes”, “no” or “more or less” and refer to the current state of respondents’ lives. A loneliness scale score ranging from 0 (not lonely) to 6 (intensely lonely) was derived by summing the neutral and positive answers (“more or less”, “yes”) on the negatively formulated items and neutral and negative answers (“more or less”, “no”) on the positively formulated items. The shortened De Jong Gierveld scale has been validated for a range of European countries, including France (De Jong Gierveld and Van Tilburg 2010). The internal consistency of the scale was satisfactory in our sample (KR-20 = .74).

#### Explanatory variables

The two main explanatory variables in our models are parental health limitations and daughters’ provision of care to the widowed parent. Health limitations are defined here as limitations in the ability to perform everyday activities due to health problems. A parent was coded as having a health limitation if the daughter reported that the parent was limited in her/his ability to perform everyday activities because of a physical or mental health problem or disability.

The type of caregiving considered is provision of personal care, e.g. help with dressing or bathing (cf. Amirkhanyan and Wolf 2003, 2006; Hansen and Slagsvold 2015; Hansen et al. 2013; Wolf et al. 2015). Providing personal care is more intensive and less routine than help with, for instance, shopping or household chores (Walker et al. 1995), and might therefore be expected to be more stressful and more strongly linked to feelings of loneliness (cf. Hansen and Slagsvold 2015; Pearlin et al. 1990). Respondents were asked: “During the last twelve months, have you helped someone regularly with personal care tasks, for example, with eating, getting out of bed, getting dressed, bathing, or going to the toilet?”. Respondents who reported providing care were then asked to name up to five persons to whom they provided help with personal care tasks. Daughters were coded as care providers if they reported having provided help with personal care tasks and mentioned the widowed parent as a recipient of this help.

#### Control variables

Age, partnership status, the presence of children in the household and employment status are time varying characteristics associated with care provision (Grundy and Henretta 2006; Lee et al. 2015; Leopold et al. 2014), as well as with loneliness (Lauder et al. 2004; Pinquart and Sörensen 2001; Theeke 2009; Van den Broek and Grundy 2017). We therefore included the daughter’s age and dichotomous variables capturing whether the daughter was living with a partner, the presence of the daughter’s children in her household, and whether she was employed.

Given that depression and loneliness are closely linked (Cacioppo et al. 2006), we also estimated models in which we added depressive symptoms as a robustness check. Depressive symptoms were measured with the 7-item depressed affect subscale of the 20-item Center for Epidemiologic Studies Depression (CES-D) scale (Hansen and Slagsvold 2011; Radloff 1977). For each of the seven statements, e.g. “I felt sad”, respondents were asked how frequently they had experienced it during the week prior to interview. Each item used a 1–4 response scale ranging from seldom or never to most or all of the time. Item scores were summed to give an overall score ranging from 7 to 28. Reliability analysis indicated that the internal consistency of the summed scale was high in our sample (Cronbach’s *α* = 0.91).

### Statistical analysis

We estimated fixed effects linear regression models to study intra-individual change in daughters’ feelings of loneliness. In longitudinal fixed effects models, within-person means over time are subtracted from scores in each observation for both outcome and explanatory variables. Consequently, all time-invariant characteristics, including those not observed, are accounted for and omitted variable bias issues are limited to time varying factors (Allison 2009). In our models, change in daughters’ feelings of loneliness is thus related to temporal variation within persons in the explanatory variables. We account for potential heteroscedasticity due to the panel nature of our data by estimating our models with cluster-robust standard errors (White 1980).

We present two models. In the first model, change in feelings of loneliness is regressed on change in parental health limitations and in the time varying control variables listed earlier. In the second model, daughters’ provision of personal care is added. This addition allows us to assess whether parental health limitations are associated with daughters’ feelings of loneliness regardless of whether or not daughters provide personal care, and whether there is an effect on loneliness of care provision that is not confounded by parental health limitations.

## Results

Descriptive statistics for our sample are presented in Table 1. The average loneliness score on the short De Jong Gierveld Loneliness scale was 1.6, which is very similar to scores reported by De Jong Gierveld and Van Tilburg (2010) for the general French population aged 18–59. In 4.5% of the observations the daughters provided personal care to the widowed parent. This may appear low, given that in one third of the observations the widowed parent had health limitations. However, parents may have been able to draw on help from other sources, including from other children and from publicly provided long-term care services. Furthermore, a limitation in the ability to perform everyday activities does not necessarily imply a need for personal care, such as help with dressing or bathing (cf. Wolf et al. 2015).

**Table 1:** Descriptive statistics: means, percentages

Continuous variables	Mean score	SD
Loneliness	1.6	1.7
Age	49.4	9.9

Categorical variables	Percentage	Number of transitions^a^
Parent has health limitation	33.2	191
Provides personal care to parent	4.5	64
Lives with partner	76.5	72
Children in household	55.9	96
Employed	61.7	154
Parent sex		
Female	88.5	
Male	11.5	

Data are from Etude des relations familiales et intergénérationnelles (ERFI), waves 1–3; weighted; number of observations: 1485, number of daughters: 557

^a^Number of transitions refers to within person changes on categorical explanatory variables

Results of our fixed effects regression analyses are presented in Table 2. Consistent with our first hypothesis, the first model showed that parental health limitations were associated with raised feelings of loneliness among their daughters. Consistent with earlier work (e.g., Theeke 2009), the model furthermore indicated that the presence of a spouse or partner was protective against loneliness. No significant effects of change in employment status or in the presence of children in the household on change in daughters’ feelings of loneliness were found.

**Table 2:** Results from fixed effects regression models of daughters’ loneliness

	Model 1		Model 2	
	*b*	(95% CI)	*b*	(95% CI)
Health limitations parent	0.37**	(0.13; 0.61)	0.34**	(0.09; 0.59)
Care provision			0.40	(− 0.21; 1.01)
Age	− 0.02	(− 0.05; 0.01)	− 0.02	(− 0.05; 0.01)
Lives with partner	− 0.39*	(− 0.78; − 0.01)	− 0.41*	(− 0.79; − 0.02)
Children in household	− 0.22	(− 0.52; 0.07)	− 0.24	(− 0.54; 0.05)
Employed	0.00	(− 0.25; 0.26)	0.01	(− 0.24; 0.26)
Bayesian Information Criterion (BIC)	3861.4		3862.4	

Data are from Etude des relations familiales et intergénérationnelles (ERFI), waves 1–3; weighted; number of observations: 1485; number of daughters: 557; cluster-robust standard errors

*p < .05; **p < .01

The provision of personal care was added in the second model. A comparison of the Bayesian Information Criterion (BIC) statistics of the first and the second model indicated that this addition did not improve the model fit (cf. Schwarz 1978). The magnitude of the coefficient estimate for the effect of care provision was very similar to the magnitude of the coefficient estimate for the effect of parental health limitations. The effect of care provision on daughters’ feelings of loneliness was not statistically significant, however. Our analyses thus did not provide support for our second hypothesis that the provision of personal care to widowed parents is associated with raised feelings of loneliness. The inclusion of personal care provision did not substantially attenuate the effect of parental health limitations found in the first model. The effect of parental health limitations also remained significant, suggesting that parental health limitations affect daughters’ feelings of loneliness regardless of whether or not daughters provide personal care to the parent.

Given that depression and loneliness are closely linked (Cacioppo et al. 2006), we performed a robustness check, whereby we re-estimated the models presented in Table 2 and added depressive symptoms as an explanatory variable. The results (available as online supplement) indicated that, although a strong association between depressive symptoms and loneliness was found, the effect of parental health limitations on loneliness remained statistically significant and the point estimate remained virtually unchanged. The impact of changes in parental health limitations on changes in daughters’ feelings of loneliness was thus not attributable to changes in depressive symptoms. Also, the point estimate of the (non-significant) effect of care provision did not change substantially when depressive symptoms were added to the model.

## Discussion

Loneliness research has primarily focused on the role of personal characteristics and circumstances, such as gender, age, marital and employment status, health, and socio-economic resources (Ong et al. 2016; Pinquart and Sörensen 2001; Routasalo and Pitkala 2003). Building on the notion from the life course perspective that human lives are linked, the current study assessed how daughters’ feelings of loneliness are impacted when widowed parents develop health limitations, and when daughters take on personal care tasks in response. We used longitudinal French data on daughters with a widowed parent to assess (a) whether having a widowed parent with a health limitation were associated with daughters’ feelings of loneliness regardless of whether or not the daughter provided personal care, and (b) whether there was an effect of providing personal care for a widowed parent on daughter’s loneliness not accounted for by the parental health limitations.

As hypothesized, the analyses showed that parental health limitations were associated with raised feelings of loneliness among daughters of widowed parents, regardless of whether or not the daughters provided care. It has been argued that having a parent in need of care is a stressful experience for adult children, regardless of whether they provide care (Amirkhanyan and Wolf 2003, 2006). Children may be more likely to experience a discrepancy between the support desired and the support received from their personal network of relationships when their parents develop health limitations, and this may result in raised feelings of loneliness. No significant effect of care provision was found.

The current study has some limitations. As described earlier, in our health limitations measure it was not specified exactly which everyday activities parents were unable to perform. Therefore, we did not know whether or not a widowed parent with health limitations in our sample was in need of personal care, such as help with dressing or bathing, which was the type of caregiving considered. Also, daughters who did not provide personal care may have provided other forms of support, such as accompanying the parent to doctor appointments or helping with grocery shopping.

We did not find a detrimental effect of providing personal care to parents on loneliness among daughters. However, this is not the same as demonstrating that no such relationship exists. If caregiving tasks lead adult children to neglect their personal social networks, as some qualitative studies suggest, then this may result in a deterioration of the quality of these networks, but plausibly only after a certain period of time. Effects of caregiving on loneliness may thus only emerge after a lag, as is the case with health effects of caregiving (Coe and Van Houtven 2009). Our data only allowed us to determine whether or not the daughter had provided frequent personal care to the parent in the twelve months prior to the interview. Data were collected at three-year intervals, so we do not know how long daughters had been providing care. Also, we do not know whether daughters who had provided frequent personal care to the parent in the twelve months prior to the interview were still care providers when they were interviewed. Moreover, our care measure did not capture the intensity of the care provided. This is unfortunate, because research has shown that caregiving is considered more burdensome when more hours per week are spent on caregiving. Future studies with more detailed measures of care provision than the study used here might find that associations between caregiving and loneliness depend on the intensity of care provision.

The fact that we found no significant effect of care provision on loneliness—even though the magnitude of the coefficient estimate for the effect of care provision was substantial—might also reflect the limited statistical power of the analysis. In the sample we used, there were only 64 transitions to or from the caregiver role. This relatively low number of transitions implies that the risk of making a type II error (not rejecting the null hypothesis when an effect exists in the population) may be substantial, particularly given that the (unknown) effect in the population may be small.

To our best knowledge, the current study constitutes the first attempt to disentangle the impact of parental health limitations and of the provision of care to ageing parents on loneliness among adult children. As described earlier, we restricted our sample to daughters of widowed parents for theoretical and pragmatic reasons. This obviously limits the extent to which our findings can be generalized. More research is needed to investigate the links between health limitations of non-widowed older parents and feelings of loneliness among their children. For instance, having two parents that are both in need of help may be even more stressful than having a widowed parent with care needs. Moreover, antecedents of loneliness are known to differ between men and women (Dykstra and De Jong Gierveld 2004; Van den Broek 2017). Future studies drawing on larger samples including substantial numbers of sons who transition to or from the role of caregiver are needed to assess whether the impact of widowed parents’ development of health limitations and of providing care to parents is similar for sons as reported here for daughters.

The results presented here raise the question how changes in the health limitations of widowed parents may lead to changes in the quality of life of their daughters. Health limitations are known to make older parents less likely to provide support to adult children (Albertini et al. 2007). Drawing mostly on qualitative research, we furthermore argued that the stressful experience of seeing one’s parent with health limitations might increase daughters’ need and desire for a supportive network. When their actual social networks are insufficiently supportive, then this might result in raised feelings of loneliness. Future research is needed to test whether these supposed mechanisms effectively underlie our finding that among daughters of widowed parents in France parental health limitations are associated with raised feelings of loneliness, regardless of whether or not daughters provide care.

Despite the limitations of the current study, the finding that parental health limitations are—at least among daughters of widowed parents in France—associated with raised feelings of loneliness regardless of whether or not the daughter provides care in our opinion constitutes a valuable contribution to the literature. Prior research, both qualitative and quantitative, on the impact of health limitations of older parents on the lives of their adult children has focused mostly on issues related to informal caregiving. Our findings, as well as those of a small number of studies about depressive symptoms (Amirkhanyan and Wolf 2003, 2006; Wolf et al. 2015), suggest that more attention to the psychosocial impact of parental health limitations—net of actual caregiving—on adult children’s lives is warranted.

## Electronic supplementary material

Below is the link to the electronic supplementary material.

Supplementary material 1 (DOCX 38 kb)
