# Supplementary material for: Parental health limitations, caregiving and loneliness among women with widowed parents: longitudinal evidence from France
Source: Eur J Ageing. 2018 Feb 12;15(4):369–77. doi: 10.1007/s10433-018-0459-2 (PMC6250644; doi:10.1007/s10433-018-0459-2)
Supplement: Supplementary file 1 — Supplementary material 1 (DOCX 38 kb) [file 10433_2018_459_MOESM1_ESM.docx]

Online Supplement Table A. *Descriptive statistics: means, percentages (not weighted)*

| *Continuous variables* | Mean score | Standard deviation |
| --- | --- | --- |
| Loneliness | 1.5 | (1.7) |
| Age | 49.5 | (9.9) |
|  |  |  |
| *Categorical variables* | Percentage | Number of transitions^1^ |
| Parent has health limitation | 31.6 | 191 |
| Provides personal care to parent | 5.1 | 64 |
| Lives with partner | 71.3 | 72 |
| Children in household | 52.3 | 96 |
| Employed | 65.9 | 154 |
| Parent sex: |  |  |
| Female | 88.7 |  |
| Male | 11.3 |  |

Notes: *Data are from Etude des relations familiales et intergénérationnelles (ERFI), Waves 1-3; Not weighted; Number of observations: 1,485, Number of daughters: 557; ^1^ Number of transitions refers to within person changes on categorical explanatory variables*

Online Supplement Table B. *Results from fixed effects regression models of daughters’ loneliness (not weighted)*

|  | Model 1 | | Model 2 | |
| --- | --- | --- | --- | --- |
|  | b | (95% CI) | b | (95% CI) |
|  |  |  |  |  |
| Health limitations parent | 0.34** | (0.12; 0.57) | 0.32** | (0.09; 0.55) |
| Care provision |  |  | 0.19 | (-0.16; 0.55) |
| Age | -0.01 | (-0.04; 0.01) | -0.01 | (-0.04; 0.01) |
| Lives with partner | -0.28 | (-0.64; 0.07) | -0.29 | (-0.64; 0.06) |
| Children in household | -0.21 | (-0.48; 0.05) | -0.22 | (-0.49; 0.05) |
| Employed | -0.01 | (-0.25; 0.22) | -0.02 | (-0.25; 0.22) |
|  |  |  |  |  |
| Bayesian Information Criterion (BIC) | 3,798.4 |  | 3,804.0 |  |

Notes: *Data are from Etude des relations familiales et intergénérationnelles (ERFI), Waves 1-3; Not weighted; Number of observations: 1,485; Number of daughters: 557; cluster robust standard errors;* * *p < .05; ** p < .01*

Online Supplement Table C. *Results from fixed effects regression models of daughters’ loneliness (adjusted for depressive symptoms)*

|  | Model 1 | | Model 2 | |
| --- | --- | --- | --- | --- |
|  | b | (95% CI) | b | (95% CI) |
|  |  |  |  |  |
| Health limitations parent | 0.37** | (0.13; 0.60) | 0.33** | (0.09; 0.57) |
| Care provision |  |  | 0.44 | (-0.10; 0.99) |
| Age | -0.01 | (-0.04; 0.02) | -0.01 | (-0.04; 0.01) |
| Lives with partner | -0.03 | (-0.42; 0.36) | -0.04 | (-0.43; 0.35) |
| Children in household | -0.18 | (-0.45; 0.10) | -0.20 | (-0.47; 0.07) |
| Employed | 0.00 | (-0.25; 0.25) | 0.01 | (-0.24; 0.25) |
| Depressive symptoms | 0.10*** | (0.08; 0.13) | 0.10*** | (0.08; 0.13) |
|  |  |  |  |  |
| Bayesian Information Criterion (BIC) | 3,725.6 |  | 3,724.3 |  |

Notes: *Data are from Etude des relations familiales et intergénérationnelles (ERFI), Waves 1-3; Weighted; Number of observations: 1,485; Number of daughters: 557; cluster robust standard errors;* * *p < .05; ** p < .01; *** p < .001*
